# Nudging for others’ sake: An ethical analysis of the legitimacy of nudging healthcare workers to accept influenza immunization

**DOI:** 10.1111/bioe.12819

**Published:** 2020-10-13

**Authors:** Mariette van den Hoven

**Affiliations:** ^1^ Utrecht University Ethics Institute Utrecht The Netherlands

**Keywords:** ‘as judged by themselves’ standard, beneficence, harm, influenza immunization of healthcare personnel, nudging, other‐regarding nudges, solidarity

## Abstract

A core idea underlying nudging is that it helps individuals to achieve their own goals, yet many nudges actually aim at collective goals or specifically target the benefit of others. An example is nudging healthcare workers to be vaccinated against influenza. I distinguish between self‐regarding nudges, which primarily benefit the nudgee, and other‐regarding nudges, which mainly benefit others, and argue that the default justificatory reason to legitimize self‐regarding nudges, namely the ‘as judged by themselves’ standard, does not apply and that we need to look for other justifications. I examine several possible moral justifications to support strong other‐regarding nudges, namely beneficence, the harm principle and solidarity.

## 1 INTRODUCTION

A core idea underlying nudging is that it helps individuals to achieve their *own* goals.^1^ Nudges can help people to adopt a healthier lifestyle, to establish a good pension plan, or to behave more sustainably, but only insofar as they already desire to live a healthier life, to have a good pension plan or to live more sustainably. Nudges are self‐regarding if they aim primarily to benefit the nudgee. There are, however, multiple examples of nudges that do not aim to benefit nudgees but instead focus on collective aims or on accruing benefit to others. Such nudges, I will argue, should be conceived as other‐regarding nudges. I distinguish between weak other‐regarding and strong other‐regarding nudges.

The legitimacy of nudges in general has been frequently debated,^2^ and nudges have been criticized for being manipulative and disrespectful of the autonomy of individuals.^3^ So, when presenting the concept of nudging, Thaler and Sunstein felt obliged to offer justificatory reasons to legitimize changing the choice architecture. They presented the ‘as judged by themselves’ standard, affirming that the autonomy of individuals must always be respected and protected by choice architects.^4^ In this paper, I will show that this standard does not properly fit nudges that aim to benefit others and that we need to seek other considerations to justify these types of nudges. The concepts of harm, benefit and solidarity may support the legitimacy of these other‐regarding nudges.

In the next section, I will first present the case of influenza immunization as an example of other‐regarding nudges and explain why nudges that aim to stimulate the immunization of healthcare workers should be thought of as other‐regarding nudges. In Section 3, the ‘as judged by themselves’ standard is presented.^5^ It will become clear that it is difficult to apply this standard to other‐regarding nudges, so these interventions need a different ethical justification. I then distinguish between weak and strong other‐regarding nudges, and in Section 4 I examine several justificatory reasons that may support strong other‐regarding nudges. Finally, in Section 5, various other‐regarding nudges that are currently being used in practice are evaluated, and conditions to determine the legitimacy of specific nudges are discussed.

## 2 INFLUENZA IMMUNIZATION OF HEALTHCARE WORKERS AND NUDGING

For more than a decade, the Center for Disease Control (CDC) has recommended the influenza immunization of healthcare workers as well as the immunization of patients who are at risk of suffering serious consequences from the influenza virus. The CDC states that ‘by getting vaccinated, you help protect yourself, your family at home, and your patients’.^6^ The World Health Organisation (WHO) states that healthcare workers should be immunized to protect not only others but also themselves from contracting flu.^7^ Protecting frail patients is an important reason to offer influenza immunization, as healthcare workers in healthcare facilities can easily become vectors for spreading viruses within these institutions. Influenza illness can cause pneumonia and other serious effects in (frail) patients.^8^ Alongside the immunization of patients, the immunization of healthcare workers can offer additional protection to patients, so that herd immunity can be achieved.^9^ In contrast to the advice of the WHO and CDC, however, the Dutch Health Council^10^ concluded in 2007 that the vaccination of healthcare workers relies mainly on an other‐regarding consideration to be immunized: it primarily helps to protect the health and wellbeing of vulnerable patients. The Council therefore recommends that, in particular, professional healthcare workers who are in close patient contact should take responsibility and be immunized. It seems clear that in their role as professional healthcare workers, the importance of protecting frail patients is a decisive consideration in the decision to advice immunization. Thus, protecting frail patients is presented as an other‐regarding benefit. The fact that employees also protect themselves and their families is an additional benefit.

The average uptake of influenza vaccines amongst healthcare professionals is on average less than 30% in European countries, but it is much higher in countries such as the United States or Australia.^11^ It seems that healthcare workers are not always motivated to actually receive influenza vaccines. Given such low rates of uptake, the question of whether to introduce mandatory immunization naturally arises. In some countries, such as the Netherlands, however, voluntary immunization has been quite successful for child immunization programs,^12^ and the chances of introducing mandatory immunization programs are quite low. So, in countries where mandatory immunization is less likely to be accepted, soft measures to increase immunization rates seem a good alternative.^13^ Nudges are a popular tool to stimulate influenza immunization uptake, and a variety of nudges are currently used in the work place: sending reminders, using peer pressure from colleagues, or making default appointments to get a flu shot. Some institutions present departmental statistics or offer rewards if immunization rates increase. A ‘red dot’ campaign has been suggested, asking immunized staff to wear a red sticker on their nametag.^14^ Some nudges have a gamifying aspect, for example offering awards or cake for the best‐performing division.

Two points should be noted with regard to nudges to stimulate influenza vaccination. First, as the vaccination of healthcare professionals is motivated primarily by the benefit accruing to others, nudges to stimulate uptake could also be seen as other‐regarding. Second, some nudges seem, intuitively, more appropriate than others. We will first discuss the legitimacy of nudges and then turn to the question of whether all kinds of other‐regarding nudges are morally justified.

## 3 ‘AS JUDGED BY THEMSELVES’ STANDARD

Much ethical debate has arisen around the question of whether governments or public organizations should be allowed to use nudge interventions to steer the behaviour of people. For some, the thought that governments use behavioural insights to steer the behaviour of citizens is troubling and goes against the liberty of individuals.^15^ Others, such as Peter John, have argued that this fear is misplaced and that we should focus on establishing good public policies instead of possible infringements on autonomy: ‘Policy makers may adopt them [i.e. public policies] if they are authorized correctly, have evidence behind them, are evaluated properly and where any potential negative effects are outweighed by the benefits, so long as individual rights are not violated.’^16^ Some agree with this view and claim that nudges are instruments or tools that need no special justification.^17^ There is a lot to be said in favour of this view, yet the argument does not hold if we look at specific interventions by governments. Quite often, we are faced with a trade‐off between conflicting duties, and it needs to be clear how interventions are supported by the basic principles to which the government is bound. The Nuffield Council on Bioethics has presented the ‘stewardship’ model as a means to navigate between two important principles in democratic societies, namely the freedom of citizens and the responsibility to provide the conditions in which citizens can live a healthy life.^18^ They introduced a so‐called intervention ladder from the least to the most coercive or intrusive measures: ‘the further up the ladder the state climbs, the stronger the justification has to be’.^19^ Notice that each step on the ladder, including the decision to do nothing, requires justification. The attempt of Thaler and Sunstein to offer a framework of libertarian paternalism must be evaluated in this light, as it intends to show that nudges are legitimate interventions and respect the autonomy of agents. They argue that a nudge ‘tries to influence choices in a way that will make choosers better off, as judged by themselves’.^20^ This ‘as judged by themselves’ standard is necessary to protect people from unwarranted paternalist interventions. The standard emphasizes that steering the behaviour of people is only acceptable if it aligns with the ends that individuals pursue. I will focus in more detail on this condition, which is offered to support the legitimacy of nudging.

Sunstein argues in *The ethics of influence* that the condition ‘as judged by themselves’ should be conceived as a *standard*, as a lodestar to determine the legitimacy of nudges. It protects agents from others and is a ‘reasonable test for all exercises of official power, at least when third parties are not at risk’.^21^ In many situations, it is fairly straightforward to determine another’s judgement is, but there are situations where this is less clear. On those occasions, he argues, we should aim for reflected and informed choices: ‘When there is a divergence, choice architects should follow people’s reflective judgements’.^22^ Hence, there is a clear appeal to choice architects not to rely either on the unreflective ad hoc judgements of people or on their own views. We should avoid situations where choice architects themselves decide what the good choice entails. After all, there is a clear danger that individuals will be perverted via nudges to ex‐post embrace the aims of a nudge. With this claim, Sunstein clearly rejects perfectionist views on ‘the good life’ and embraces the view that we should be led by the actual choices of people, even if these are wrong in the eyes of others.^23^ The ‘as judged by themselves’ standard thus focuses on the preferences (first‐ or second‐order preferences) of individuals and stresses the relevance of informed choice.

### 3.1 Other‐regarding nudges and the standard

The standard fits well with many self‐regarding nudges, i.e., with nudges that mainly benefit the ends and choices of individuals. Will it also support other‐regarding nudges? Karin Yeung points out that ‘many of the proposals advocated in *Nudge* are concerned with shaping *other‐regarding* decisions in order to promote collective welfare rather than with “improving” an individual’s *self*‐*regarding* actions’.^24^ For example, the famous ‘fly in the urinary’ at Schiphol Airport will not immediately benefit the user himself, but rather subsequent travellers. It might not be an immediate preference or end of individuals to not spoil toilets; the main benefit will accrue to others. In other words, it is possible to distinguish between the ends of individuals and the benefit of a nudge intervention. Many of my actions will not directly benefit me, but I still choose to do them. If I desire to promote what is good for others, the benefit others experience from my actions can still be explained by the ‘as judged by themselves’ standard. In the example of the urinary, we share the collective aim to not pollute public toilets. Will the standard apply to all cases of other‐regarding nudges? To answer this question, I suggest that we need to distinguish among three types of nudges. First, there are self‐regarding nudges, which primarily focus on the ends and choices of individuals and the benefit to themselves. Next, we can distinguish two types of other‐regarding nudges, namely weak and strong other‐regarding nudges. I will, in line with the definition used by Ismaili M’hamdi and co‐workers, define weak other‐regarding nudges as: ‘when the principal but not necessarily sole beneficiary of the nudge is not the nudgee’ but others.^25^ Thus, the principal consideration is to benefit others. Strong other‐regarding nudges are those nudges for which the sole beneficiary of the nudge is not the nudgee. These benefits to others can, in extreme versions, override the ends of nudgees. Then, the choices and ends of individuals become irrelevant because of the greater good that we try to achieve,^26^ or because the benefits to others can trump the interests of agents, for example to prevent serious harm. An example used by Ismaili M’hamdi and co‐workers is the prevention of alcohol use by pregnant women. A strong other‐regarding nudge would give priority to the interests of the unborn child, irrespective of the actual desires and ends of the mother‐to‐be. In many situations, however, pregnant women will desire a healthy child, and nudging them towards an alcohol‐free pregnancy can be supported by weak other‐regarding nudges.

With regard to nudges, there is a continuum between self‐regarding nudges and strong other‐regarding nudges. Most nudges lie somewhere between the extremes, and their justification will depend on the context. For example, a default registration as an organ donor will be situated on the other‐regarding side of the spectrum, while reminders to register as an organ donor will tend to be situated more towards the self‐regarding pole. The justification of nudges can also depend on the actual choices and preferences of nudgees, combined with the expected benefit. If healthcare workers agree that the protection of frail patients is necessary, but fail to be immunized, a nudge can help them to achieve their own ends as well as benefit their patients. However, for those healthcare workers who are not convinced that immunization is necessary, for which low immunization rates could be a clear indication, nudging will rely less on their own actual choices and ends. In this case, nudging healthcare professionals to benefit frail patients becomes a strong other‐regarding nudge, since in this case the ends and choices of nudgees are considered less relevant than the benefits to frail patients.^27^ Strong other‐regarding nudges can, ultimately, trump the ends and preferences of individuals. If we deem nudges a necessary means to stimulate immunization, justify the use of nudges. I sugg Vaccination against classical est that we look for justificatory reasons that support strong other‐regarding nudges, as these are at the extreme opposite of the ‘as judged by themselves’ standard and find no support in it.

## 4 THE LEGITIMACY OF OTHER‐REGARDING NUDGES

What considerations could justify the use of strong other‐regarding nudges? In this section, several criteria will be presented that could serve as legitimization for other‐regarding nudges. First, we will look at the principle of beneficence, then at the harm principle, and finally at the concept of solidarity. The case of influenza immunization for healthcare workers will serve as a reference point. I will focus on the question of whether the proposed candidate principles are necessary and sufficient conditions to support strong other‐regarding nudges.

### 4.1 Beneficence

It seems obvious to think of the principle of beneficence to support strong other‐regarding nudges. In the field of bioethics, this principle is widely embraced and has strong appeal. Thaler and Sunstein, when discussing the case of organ donation, also seem to assume a moral principle of beneficence: ‘libertarian benevolence is put forward as justification’, because the availability of ‘this valuable good fully depends on the willingness of others to donate’.^28^ In our case, the benefit for frail patients not to suffer from the consequences of influenza is dependent on the ability to establish herd immunity. For this purpose, staff need to be immunized. Ismaili M’hamdi and co‐workers argue that beneficence might not be a sufficient condition to justify other‐regarding nudges, because of the nature of the principle of beneficence: it is not always immediately clear if beneficence is to be seen as an ethical *principle*, including duties of beneficence, or if it is merely a moral *ideal*.^29^ Thus, ‘the fact that we are dealing with (1) valuable goods whose (2) availability depends on the willingness of others to provide them and (3) these others have an interest in providing these goods provide sufficient justification for OG’ (equivalent to other‐regarding nudges to promote the good) will not be decisive, because there can always be reasons that trump the promotion of the good of others.^30^ Beneficence does not, in general, always lead to a clear moral duty. Such duties are only clear in rescuing another’s life or when we can prevent harm from occurring. Thus, only specific actions that affect specific people are obligatory, while other acts of beneficence, especially those targeting a larger group, are generally not obligatory. Is there a strict duty of beneficence in the case of influenza immunization? In favour of such a strict duty we could state that, from the perspective of professional health workers, protection against influenza is a specific action that targets the collective in healthcare organizations. However^31^, protection against influenza can be achieved in multiple ways, for example by staying home when feeling ill^32^ or wearing a face mask. It is therefore difficult to consider non‐immunization as morally reprehensible if alternatives are available. Furthermore, immunization infringes upon a person’s bodily integrity and can be considered as more demanding than other professional responsibilities such as wearing a uniform or taking hygiene measures.^33^ So, beneficence in general can certainly be used as a justificatory ground to prevent influenza outbreaks, but it does not suffice as the only consideration to stimulate influenza vaccination uptake, and hence cannot be used to support nudging interventions with this aim. I agree here with Ismaili M’hamdi and co‐workers that beneficence offers a necessary but not sufficient justification for other‐regarding nudges. On the other hand, one could claim that we may not need to look for moral duties of beneficence to justify nudges towards healthcare staff: all we need is a moral consideration supporting using mild interventions such as nudges. Could beneficence be applicable? Remember that we are not ruling out that beneficence can play a role, but because we are looking for moral considerations that could possibly trump the ends and choices of nudgees (i.e., support for strong other‐regarding nudges), beneficence might not provide a strong enough basis for the use of nudges to support influenza vaccination uptake amongst healthcare staff.

### 4.2 Harm

Another obvious candidate to justify other‐regarding nudges is the harm principle, originating from John Stuart Mill. Mill defends the position that the only consideration that justifies taking action against one’s will is to protect against harm happening to others. Thus, if one’s actions can seriously harm others, this warrants protection of those others.^34^ In the context of public health, the harm principle has been used widely to support interventions. Looking at other‐regarding nudges, we can see how a pregnant woman can be limited in her decisions and actions if she ‘places the future child at substantial risk of serious harm. Only then, the most effective and least intrusive interventions are justified’.^35^ The harm that can be prevented by stopping an addicted pregnant woman from using alcohol seems obvious. However, Holtug points out that the harm principle, as presented by John Stuart Mill, only justifies *coercion of the individual* in order to prevent harm.^36^ Coercion is a strong intervention that might not apply to nudges, because it is easy to resist such interventions.^37^ Krom also argues that ‘public health interventions that do not restrict the liberty of individuals in any meaningful sense, such as encouraging individuals to take part in vaccination programmes, do not require justification via the harm principle’.^38^ It seems that there is good reason to accept that the nature of nudges does not fit within the scope of the harm principle, as most nudges are mild interventions that are easily resistible and liberty‐preserving. At the same time, non‐maleficence is an appealing consideration, and Ismaili M’hamdi and co‐workers argue that the duty to avoid inflicting harm or putting people at risk is indeed a good justificatory reason to support other‐regarding nudges, for two reasons. First, harm, like illness, is often caused not by a single event but by a lifestyle pattern. Second, the ethical principles of proportionality and subsidiarity urge us to seek the mildest possible interventions that will be effective. Nudges therefore seem a good instrument to prevent the harm that can occur as a result of lifestyle pattern. ‘OH [other‐regarding nudges to prevent harm] are justified when the causes of harm viewed separately are morally wrong but not to the extent that they justify and warrant coercive interventions. In these cases, the harm principle offers necessary and sufficient justification for OH’.^39^ The idea is basically that in preventing harm we could also turn to mild interventions such as nudging if these are effective. The principles of proportionality and subsidiarity actually urge us to turn to the least intrusive means to achieve the good.

If we try to apply this line of reasoning to the case of the influenza immunization of healthcare workers, we immediately see that influenza illness is not a lifestyle issue. Instead, we are confronted with a collective action problem, namely that the cooperation of all is necessary to offer extra protection to frail patients. It is argued that protecting frail patients against influenza does lead to a prima facie duty for healthcare professionals not to inflict harm on their patients, which can outweigh other considerations.^40^ At the same time, it is recognized that alternative measures, for example hygiene measures such as wearing a face mask or meticulous hand disinfection, could also be sufficient to meet this prima facie duty.^41^ A complicating factor in our case is that, in contrast to the pregnancy example, the contributions of individual members of staff to outbreaks cannot be fully determined, because outbreaks can occur even when sufficient numbers of staff are immunized. Therefore, it might be prudent to focus on the *prevention* of harm instead of on *inflicting* harm: healthcare workers can contribute to the prevention of patients becoming ill. Prevention of harm, however, is often seen as part of the principle of beneficence and not as part of nonmaleficence.^42^ This would leave us with the conclusion that it is also a necessary, but not sufficient, consideration to support strong other‐regarding nudges.

In sum, not inflicting harm is a very strong moral consideration, but the harm principle might not apply because of the nature of nudges. Furthermore, if we were to accept the view that there is a prima facie moral duty of healthcare workers to prevent harm to frail patients, this will lead us either back to the principle of beneficence, or to a similar conclusion to with the principle of beneficence, namely that influenza immunization is not the only way to prevent harm.

### 4.3 Solidarity and professional solidarity

A third option to justify strong other‐regarding nudges is to focus on the issue from a different perspective, namely by looking at the concept of solidarity. Solidarity could be conceived as ‘shared practices reflecting a collective commitment to carry “costs” (financial, social, emotional or otherwise) to assist others’.^43^ Solidarity reminds us to take into account that humans are not solipsistic beings, but live and interact with others.^44^ Considerations of solidarity stimulate us to contribute to the overall health of society, not only our own, because we are urged to actively engage with others.^45^ Dawson and Verweij distinguish between two types of solidarity, namely rational solidarity and constitutive solidarity.^46^ Rational solidarity is a way of ‘standing together’, for example when fighting a threat. In this case, individuals can be asked to set aside their own short‐term interests for long‐term individual and collective benefits. A good example in the field of public health is a pandemic, which requires individuals to radically change their behaviour. Constitutive solidarity, on the other hand, is a social concept, which revolves around shared values, meaning and identity. People do not have to consent explicitly to this type of solidarity, as it is embedded in communities. Examples offered are the collective actions to find a missing child, the introduction of traffic calming in a neighbourhood, or improving the nutritional value of meals at local schools.^47^

Solidarity is suggested to be complementary to the dominant concept of autonomy in healthcare. Improving the health of others is not always based on self‐interest or on the harm principle. Rather, we could act out of concern for others. Solidarity stimulates ‘a gestalt shift from seeing health as personal achievement or as a matter of biological lottery to seeing health (and illness) as something mutual, something that creates responsibilities of care and concern incumbent on us all’.^48^ Taking the perspective of solidarity as justificatory reason to support strong other‐regarding nudges has as a clear advantage that the concept itself already incorporates the idea that the benefit to others could prevail and could urge us to act on behalf of those benefits. Could it be a necessary and sufficient condition? It seems that solidarity could indeed be a necessary condition, as it makes a moral appeal to individuals to benefit others for their own sake, but is it also a sufficient condition? Will solidarity not suffer from the same weakness as the principle of beneficence, in that it can be executed in multiple ways? Moreover, the debate on the concept is ongoing, and some argue that it is not a self‐standing principle, but a complementary or background consideration.^49^ I pointed out earlier that it is unclear if the candidate moral consideration has to be a moral duty, because the harm principle would allow for coercion, and hence be too strong for the type of intervention that we seek moral support for. It would therefore require further deliberation to determine if a status weaker than a moral duty would suffice to support strong other‐regarding nudges. I tend to think that it can, given the mild nature of nudge interventions.

There is one additional reason why, in the case of influenza immunization, solidarity might be a sufficient condition when combined with the notion of professionalism. Healthcare workers have a professional responsibility to protect the health and wellbeing of their patients. Adhering to hygiene measures, for example washing one’s hands or wearing a uniform, is nowadays part and parcel of one’s professional responsibilities, as is mandatory immunization against Hepatitus B.^50^ The notion of professional solidarity is used to emphasize that one is part of a community, which needs maintenance by stimulating the health and wellbeing of others without any direct self‐gain.^51^ It could be seen as a professional responsibility to be vaccinated to protect the interests of patients and even if solidarity could be supported in multiple ways, this would not exempt professionals from the responsibility to look after frail patients’ interests and be immunized. Notice that in light of this, influenza vaccination would become a professional decision, and not just a matter of personal preference or choice. One could object that not all employees in health institutions are professionals, supported by professional codes,^52^ but irrespective of professional codes of conduct, the solidarity argument supports a shared responsibility for all employees in healthcare institutions.

The idea that nudges could find support in collective considerations, like the overall good, has already been suggested by others.^53^ I think that solidarity could be a candidate justification for strong other‐regarding nudges, but that further discussion on its status is necessary. I think that it would be interesting to explore this notion further and its support for other examples of strong other‐regarding nudges.

### 4.4 Preliminary conclusion

Three types of moral considerations were explored to support strong other‐regarding nudges. Beneficence seemed a necessary but not sufficient consideration to justify nudges. The harm principle is appealing, but might not fit with the nature of nudging or might lead us back to beneficence if we focus on the prevention of harm. Harm could support weak other‐regarding nudges, when one’s own ends coincide with the benefit to others. Some healthcare professionals will accept the benefit for frail patients as a convincing consideration to accept immunization. But when health professionals do not support the aim of benefitting patients via immunization, we need stronger considerations, if we still think that nudges are a good means to stimulate the immunization of these professionals. Solidarity, starting from a collective perspective, could be a good candidate to support the use of strong other‐regarding nudges for the case of influenza immunization. It incorporates the thought that one sets aside one’s own interests and aims to support another person’s wellbeing (necessary condition), and it might indeed be that it could be a sufficient condition when combined with professional responsibility.

In the final section, I will turn to one last pressing issue, namely the question of whether the legitimacy of other‐regarding nudges will also depend on the characteristics of the nudge involved. This seems plausible, given the variety of possible nudges to stimulate influenza vaccination uptake in practice. Will all other‐regarding nudges be equally legitimate?

## 5 LIMITS TO OTHER‐REGARDING NUDGES?

As mentioned earlier, a wide variety of nudges are being used to stimulate healthcare professionals to accept immunization, including sending reminders, using peer pressure, making default appointments, giving rewards to departments, and visualization through ‘red‐dot’ campaigns. Apart from the justificatory reason why we might interfere with the liberty of individuals at all, several criteria to identify types of nudges have been presented. First, Thaler and Sunstein mention the ability to opt out of a nudge. Choice architects navigate people towards a certain decision or behaviour, but must make it easy to opt out. A red‐dot campaign makes opting out difficult, because once patients and staff know what the red dot stands for, social pressure, exclusion and stigmatization could prevent staff members from opting out. Patients could refuse to be treated by nurses who do not wear a red dot and family members of patients might accuse those without a red dot of putting their relatives at risk. Less problematic would be to sign a banner when one is immunized, as long as one’s signature was not identifiable.

Another criterion to determine the legitimacy of specific nudges is transparency. Many objections against nudging ‘are based on a fear that the underlying motivations will be illicit’.^54^ Sunstein therefore argues that ‘choice architecture should be transparent and subject to public scrutiny, certainly if public officials are responsible for it’. For him, transparency is a necessary condition in order for a nudge to be legitimate.^55^ In most situations, it is easy to inform people about the fact that they are being nudged, without affecting the effectiveness of the nudge.^56^ The transparency condition is not met when the aim of the nudge is troubled by the intervention. Suppose that the department in the hospital with the highest immunization rate is rewarded with cake or a trophy. Such nudges would bring in a game‐element, which could distract or even blind people to the purpose of the nudge. Therefore, such nudges are more problematic than nudges that clearly steer towards herd immunity, such as default appointments and peer pressure via posters.

The third criterion mentioned in this paper was an adequacy criterion.^57^ If an intervention will not be very adequate (i.e. effective), then we should seriously doubt whether that nudge is acceptable. If making default appointments to be immunized is not very effective, and is more controversial than, for example, walk‐in hours to be immunized, the latter type of nudge seems preferable, because of its adequacy. The fourth consideration is proportionality: steering people’s behaviour, especially in cases where it concerns others, should involve the least intrusive means to accomplish the desired outcome, and hence respect the autonomy of individuals as much as possible. Again, the default appointment is a more intrusive measure than the walk‐in‐hours approach to influenza immunization, and hence should be preferred from a proportionality perspective. These four criteria are, in addition to the justificatory reason to legitimize other‐regarding nudges, helpful in determining whether the type of nudge is acceptable or not. For each intervention that will steer people’s behaviour, it will depend on contextual factors if the nudges will be ultimately acceptable. A case‐by‐case evaluation of types of nudges is therefore necessary.

## 6 CONCLUSION

In this paper, I examined what motivations support the use of nudges in general and of other‐regarding nudges specifically, in order to examine the legitimacy of nudges used to stimulate the uptake of influenza immunization in healthcare workers. I distinguished self‐regarding nudges, which are the default supported by the ‘as judged by themselves’ standard, from other‐regarding nudges, which focus on the benefit to others and can overrule the ends that individuals have for themselves. For each type of nudge, a different type of justification is necessary. I examined three possible moral considerations to find support for the strong other‐regarding considerations, and concluded that beneficence and the harm principle are appealing, but have limitations. Taking a more collective approach invites us to consider solidarity as a possible moral support for the use of other‐regarding nudges. However, this does not lead to the acceptance of all other‐regarding nudges. I examined several examples of nudges used to stimulate influenza uptake and concluded that four other considerations are relevant, namely easy opt out, transparency, adequacy and proportionality. Nudging, including other‐regarding nudging, requires a careful case‐by‐case examination to determine its legitimacy.

## CONFLICT OF INTEREST

The author declares no conflict of interest.

## Footnotes

^1^
Thaler, R. H., & Sunstein, C. (2008). *Nudge. Improving decisions about health, wealth and happiness*. London, U.K.: Penguin books.

^2^
Engelen, B. (2019). Ethical criteria for health‐promoting nudges: A case‐by‐case analysis. *American Journal of Bioethics, 19*(5), 48–59; Ashcroft, R. E. (2013). Doing good by stealth: Comments on ‘Salvaging the concept of nudge’. *Journal of Medical Ethics, 39*(8), 494.10.1136/medethics-2012-10110923455009

^3^
Hausman, D. M., & Welch, B. (2010). Debate: To nudge or not to nudge. *Journal of Political Philosophy, 18*, 123–136. 10.1111/j.1467-9760.2009.00351.x; Nys, T., & Engelen, B. (2017). Judging nudging: Answering the manipulation objection. *Political Studies, 65*, 199–214. 10.1111/j.1467-9760.2009.00351.x; Vugts, A., Van den Hoven, M., De Vet, E., & Verweij, M. (2018). How autonomy is understood in discussions on the ethics of nudging. *Behavioral Public Policy, 2*, 1–16. 10.1111/j.1467-9760.2009.00351.x; Sunstein, C. (2016). *The ethics of influence* *Government in the age of behavioral science*. Cambridge, U.K.: Cambridge University Press.

^4^
Thaler & Sunstein, op. cit.

^5^
Ibid: 5.

^6^
Center for Disease Control. Retrieved from https://www.cdc.gov/flu/healthcareworkers.htm. Accessed Aug 14, 2019.

^7^
World Health Organization. Influenza vaccine protection for both health care workers and patients. Retrieved from http://www.euro.who.int/en/health‐topics/communicable‐diseases/influenza/news/news/2016/10/influenza‐vaccine‐protection‐for‐both‐health‐care‐workers‐and‐patients. Accessed Aug 14, 2019.

^8^
Jefferson, T., Rivetti, D., Rivetti, A., Rudin, M., Di Pietrantonj, C., & Demicheli, V. (2005). Efficacy and effectiveness of influenza vaccines in elderly people: a systematic review. The Lancet, 366,(9492), 1165–1174. 10.1016/s0140-6736(05)67339-4 16198765

^9^
Carman, W. F., Elder, A. G, Wallace, L. A., McAulay, K., Walker, A., Murray, G. D., & Stott, D. J. (2000). Effects of influenza vaccination of health‐care workers on mortality of elderly people in long‐term care: a randomised controlled trial. The Lancet, 355,(9198), 93–97. 10.1016/s0140-6736(99)05190-9 10675165

^10^
Dutch Health Council. (2011). *Griepvaccinatie: Herziening van de indicatiestelling*. Publicatienr 2007/09. [Influenza vaccination: Revision of assessment]. Den Haag: Gezondheidsraad.

^11^
Guglielmo, D., Toletone, A., Sticchi, L., Orsi, A., Bragazzi, N. L., & Durando P. (2018). Influenza vaccination in healthcare workers: A comprehensive critical appraisal of the literature. Human Vaccines & Immunotherapeutics, *14*(3), 772–789. 10.1080/21645515.2017.1348442 PMC586178528787234

^12^
Rijksinstituut voor de Volksgezondheid (RIVM). (2018). *Vaccinatiegraad en jaarverslag rijksvaccinatieprogramma 2018* [Vaccination rates and annual report vaccination program 2018]. Report, RIVM. 10.21945/RIVM-2019-0015

^13^
Dubov. A., & Phung, C. (2015). Nudges or mandates? The ethics of mandatory flu vaccination. *Vaccine, 33*, 2530–2535.10.1016/j.vaccine.2015.03.04825869886

^14^
Ibid.

^15^
Selinger, E., & Whyte, K. (2011). Is there a right way to nudge? The practice and ethics of choice architecture. *Sociology Compass*, *5*(10), 923–935.

^16^
John, P. (2018). *How far to nudge? Assessing behavioural public policy*. Cheltenham, U.K.: Edward Elgar Publishing Inc.

^17^
Tiemeijer, W., & Anderson, J. H. (2014). Normatieve overwegingen rondom gedragssturingen [Normative considerations regarding behavioral policy]. In Raad voor de Maatschappelijke Ontwikkeling, *De verleiding weerstaan: Grenzen aan beïnvloeding van gedrag door de overheid* [Resisting the temptation: Limits to steering behaviour by governments]. Den Haag, the Netherlands: RMO.

^18^
Nuffield Council on Bioethics. (2007). *Public health: ethical issues* London, U.K. Retrieved from http://nuffieldbioethics.org/wp-content/uploads/2014/07/Public-health-ethical-issues.pdf

^19^
Ibid: vi.

^20^
Thaler & Sunstein, op. cit., p. 5.

^21^
Sunstein, C. (2016). *The ethics of influence* *Government in the age of behavioral science*. Cambridge, U.K.: Cambridge University Press, p. 43.

^22^
Ibid: 48.

^23^
Ibid: 51.

^24^
Yeung, K. (2012). Nudge as fudge. *Modern Law Review, 75*(1), 122–148.

^25^
Ismaili M'hamdi, H., Hilhorst, M., Steegers, E. A. P., & de Beaufort, I. (2017). Nudge me, help my baby: on other‐regarding nudges. Journal of Medical Ethics, *43*(10), 702–706. 10.1136/medethics-2016-103656 28122991

^26^
Nys, T. R. V., & Engelen, B. (2017) Judging nudging: Answering the manipulation objection. *Political Studies, 65*(1), 199–214.

^27^
Ahmed, F., Megan, C., Allred, N., Weinbaum, C. M., & Groshkopf (2013). Effect of influenza vaccination of healthcare personnel on morbidity and mortality among patients: Systematic review and grading of evidence. *Clinical Infectious Diseases, 58*(1), 50–57. doi: 10.1093/cid/cit580; Lam, P. P., Chambers, LW., MacDougall, D. M. P., & McCarthy, A. E. (2010). Seasonal influenza vaccination campaigns for health care personnel: Systematic review. *CMAJ*, *182*(12), E542–E548; Lemaitre, M., Meret, T., Rothan‐Tondeur, M., Belmin, J., Lejonc, J. L., Luquel, L., Piette, F., Salom, M. et al. (2009). Effect of influenza vaccination of nursing home staff on mortality of residents: A cluster‐randomized trial, *J. Am. Geriatr. Soc*, *57*(9), 1580–1586. 10.1093/cid/cit580 19682118

^28^
Ismaili M’hamdi et al., op. cit, p. 2.

^29^
Beauchamp, T., & Childress, J. (2009). *Principles of biomedical ethics* (6th ed.). Oxford, U.K.: Oxford University Press, p. 198.

^30^
Ismaili M’hamdi et al., op. cit., p. 2.

^31^
Ibid.

^32^
Harris, J., & Holm, S., (1995). Is there a moral obligation not to infect others? *BMJ, 311*, 1215–1217.10.1136/bmj.311.7014.1215PMC25511627488907

^33^
Van den Hoven, M. A., & Verweij, M. F. (2003). Should we promote influenza vaccination of health care workers in nursing homes? Some ethical arguments in favour of immunization. *Age and Ageing, 32*, 487–489.10.1093/ageing/afg08912957995

^34^
Preventing harm to others is, according to the famous quote by Mill, the only rightful justification why liberties of individuals against their will could be restricted. Otherwise the individual is sovereign.

^35^
Ismaili M’hamdi, H. et al., op. cit. p. 3.

^36^
Holtug, N. (2002). The harm principle. *Ethical Theory and Moral Practice, 5*, 357–389.

^37^
Saghai, Y. (2013). Salvaging the concept of nudge. *Journal of Medical Ethics, 39*, 487–493.10.1136/medethics-2012-10072723427215

^38^
Krom, A. (2011). The harm principle as a mid‐level principle? Three problems from the context of infectious disease control. *Bioethics*, *25*(8), 437–444.10.1111/j.1467-8519.2011.01926.x21929702

^39^
Ismaili M’hamdi et al., op. cit., p. 3.

^40^
Delden van, J. J. M., Ashcroft, R., Dawson, A., Marckmann, G., Upshur, R., & Verweij, M. F. (2008). The ethics of mandatory vaccination against influenza of health care workers. *Vaccine, 26*, 5562–5566.10.1016/j.vaccine.2008.08.00218722495

^41^
Wicker, S., Holger, F., Kempf, V.A.J., Brandt, C. (2009). Vaccination against classical influenza in health‐care workers self‐protection and patient protection. *Deutsches Årtzesblatt International, 106*(36), 567–572.10.3238/arztebl.2009.0567PMC277021019890412

^42^
Beauchamp & Childress, op. cit.

^43^
Prainsack, B., & Buyx, A. (2011). *Solidarity: Reflections on an emerging concept in bioethics,* London, U.K.: Nuffield Council on Bioethics.

^44^
Jennings, B., & Dawson, A. (2015). Solidarity in the moral imagination of bioethics. *Hastings Center Report, 45*(5), 31–38.10.1002/hast.49026332108

^45^
Jennings, B. (2015). Relational liberty revisited: Membership, solidarity and a public health ethics of place. *Public Health Ethics, 8*(1), 7–17.

^46^
Dawson, A., & Verweij, M. (2012). Solidarity: A moral concept in need of clarification. *Public Health Ethics*, *5*(1), 1–5.

^47^
Ibid: 2.

^48^
Jennings & Dawson, op. cit., p. 37.

^49^
Ibid

^50^
Van den Hoven & Verweij (2003), op. cit.

^51^
Van den Hoven, M. A., & Verweij, M. F. (2013). Professional solidarity: The case of influenza immunization. *American Journal of Bioethics*, *13*(9), 51–52. 10.1080/15265161.2013.813606; May, L. (1993). *The socially responsive self: Social theory and professional ethics*. Chicago, IL: University of Chicago Press.23952837

^52^
Antommaria, A. H. M. (2013). An ethical analysis of mandatory influenza vaccination of health care personnel: Implementing fairly and balancing benefits and burdens. *American Journal of Bioethics, 13*(9), 30–37.10.1080/15265161.2013.81473123952830

^53^
Nys & Engelen, op. cit.

^54^
Sunstein, op. cit., p. 41.

^55^
Ibid: 104.

^56^
Ibid: 119.

^57^
Ismaili‐M’hamdi et al., op. cit.

